# Zinc finger 4 negatively controls the transcriptional activator Fzf1 in *Saccharomyces cerevisiae*

**DOI:** 10.1002/mlf2.12141

**Published:** 2024-09-23

**Authors:** Ying Du, Chaoqun Ma, Stanley A. Moore, Wei Xiao

**Affiliations:** ^1^ Department of Biochemistry, Microbiology and Immunology University of Saskatchewan Saskatoon Saskatchewan Canada

**Keywords:** chemical toxicity, Fzf1, *Saccharomyces cerevisiae*, transcription regulation, zinc finger

## Abstract

Fzf1 is a *Saccharomyces cerevisiae* transcription factor containing five zinc fingers (ZFs). It regulates the expression of at least five downstream genes, including *SSU1*, *YHB1*, *DDI2/*3, and *YNR064c*, by recognizing a consensus sequence, CS2, found in these gene promoters. These gene products are involved in cellular responses to various chemical stresses. For example, *SSU1* encodes a sodium sulfite efflux protein that confers sulfite resistance. However, the underlying molecular mechanism through which Fzf1 responds to chemical stress and coordinates target gene activation remains elusive. Interestingly, several mutations in the fourth ZF (ZF4) of Fzf1 have previously been reported to confer either sulfite resistance or elevated basal‐level expression of *YHB1*, indicating that ZF4 negatively impacts Fzf1 activity. Since ZF4 is dispensable for CS2 binding in vitro, we hypothesized that ZF4 is a negative regulator of Fzf1 and that chemically induced Fzf1‐regulated gene expression occurs via de‐repression. All five genes examined were cross‐induced by corresponding chemicals in an Fzf1‐dependent manner, and all three ZF4 mutations and a ZF4 deletion conferred increased basal‐level expression and *SSU1*‐dependent sulfite resistance. A ZF4 deletion did not alter the target DNA binding, consistent with the observed codominant phenotype. These observations collectively reveal that Fzf1 remains inactive by default at the target promoters and that its activation is at least partially achieved by self‐derepression through chemical modification and/or a conformational change.

## Impact statement

This study characterized a budding yeast zinc‐finger transcription factor Fzf1 and found that it is required to activate at least five functionally unrelated genes in response to diverse chemical stresses. Surprisingly, while its N‐terminal three ZFs are required for target DNA recognition, ZF4 inhibits target gene expression in the absence of chemical stresses. Hence, the chemical activation of the target gene expression is achieved through derepression of the Fzf1 self‐inhibition. Together, this study unveils a novel regulatory mechanism by which a transcriptional factor binds its target promoter sequence in an allosterically inactive conformation by one of its inhibitory zinc fingers.

## INTRODUCTION

*Saccharomyces cerevisiae FZF1* encodes a 299 amino acid protein with five zinc finger (ZF) domains^1^. *FZF1* was first functionally identified as a transcriptional regulator of *SSU1* ^2^. Subsequent systematic studies revealed its regulatory function toward a group of genes, including *SSU1*, *YHB1*, *DDI2/3*, and *YNR064c* ^3^ *.* Notably, overexpression of *FZF1* alone is sufficient to elevate the expression of the aforementioned genes^3^.

Fzf1‐regulated genes exhibit a wide range of chemical responsiveness. For instance, *SSU1* encodes a plasma membrane protein involved in sulfite efflux^2^ and is inducible by sodium sulfite^4^. *YHB1* encodes a dioxygenase and is involved in oxidative stress responses, especially those mediated by nitric oxide (NO)^5^. *YHB1* can be induced by NO and other NO‐derived species, such as dipropylenetriamine (DPTA) NONOate, a common experimental NO donor^6^. *DDI2* and *DDI3*, two identical genes located on different chromosomes (hereafter referred to as *DDI2/3*), can be induced by the DNA‐damaging agent methyl methanesulfonate (MMS)^7^. *DDI2/3* encodes cyanamide (CY) hydratases^8^, ^9^ and can also be induced by CY^9^, ^10^. *YNR064c* encodes a protein that displays epoxide hydrolase activity in vitro^11^, although its transcriptional regulation has not yet been reported. Further investigations into *FZF1* regulation have demonstrated that deletion of *FZF1* abolishes the induction of *YHB1* by NO^3^ and the induction of *DDI2/3* by CY or MMS treatments^12^. Thus, *FZF1* functions as a positive regulator of *YHB1* and *DDI2/3* under NO and CY/MMS treatments, respectively, and potentially as a transcriptional regulator of *SSU1* under sulfite treatment conditions.

Typical ZF proteins, including Fzf1, contain a cluster of Cys_2_His_2_ (C_2_H_2_) ZFs^13^, ^14^ that coordinate a tetrahedral zinc ion via two Cys and two His residues and place an α‐helix in the major groove of duplex DNA, allowing each ZF to recognize three nucleotides^15^. Bioinformatics analysis revealed that all five Fzf1‐regulated genes share a promoter consensus sequence named CS2^3^. Deletion of CS2 from *YHB1* ^3^ or *DDI2/3* ^12^ completely abolished the chemically induced expression of these genes. Furthermore, Fzf1 specifically binds to the *DDI2/3* CS2 sequence in vitro and in vivo^12^, confirming that CS2 is the sequence‐specific target of Fzf1. However, it is unclear how Fzf1 senses a wide range of chemical stresses and becomes activated to target such functionally diverse genes.

A previous in vitro study^16^ demonstrated that the three N‐terminal zinc fingers (ZF1–3) are involved in target DNA recognition in the *SSU1* promoter, while ZF4 and ZF5 are dispensable for this activity. Interestingly, a cloned sulfite‐resistant gene *SUL1* ^17^ turned out to be allelic to *FZF1*, and the candidate mutation was found to cause an H180D amino acid substitution in ZF4^18^. Recently, a systematic mutagenesis analysis found that two other *fzf1‐ZF4* mutations, C157S and C160S, led to increased basal levels of *YHB1* expression and a lack of further induction by NO^19^, although the underlying mechanism has not been investigated.

To understand how unrelated chemical stresses activate Fzf1 and result in the induced downstream gene expression of the targeted genes, we proposed two hypotheses in this study. First, Fzf1 coordinately responds to all chemicals and thereby regulates its downstream genes. Second, Fzf1 ZF4 serves as an inhibitory component of Fzf1 to suppress its target gene expression. The observations made in this study provide experimental evidence that supports both hypotheses. The implications of this study are discussed further.

## RESULTS

### Fzf1‐regulated genes are cross‐induced by different chemicals

Our first hypothesis predicted that Fzf1‐regulated genes could be cross‐induced by different chemicals, even though these genes may not be involved in the stress response to a given chemical. Previously, it was reported that *SSU1* was inducible by sodium sulfite^16^, *YHB1* was inducible by NO^3^, and *DDI2/3* was inducible by MMS^7^ and CY^9^; however, no cross‐inducibility has been examined.

**Figure 1:**
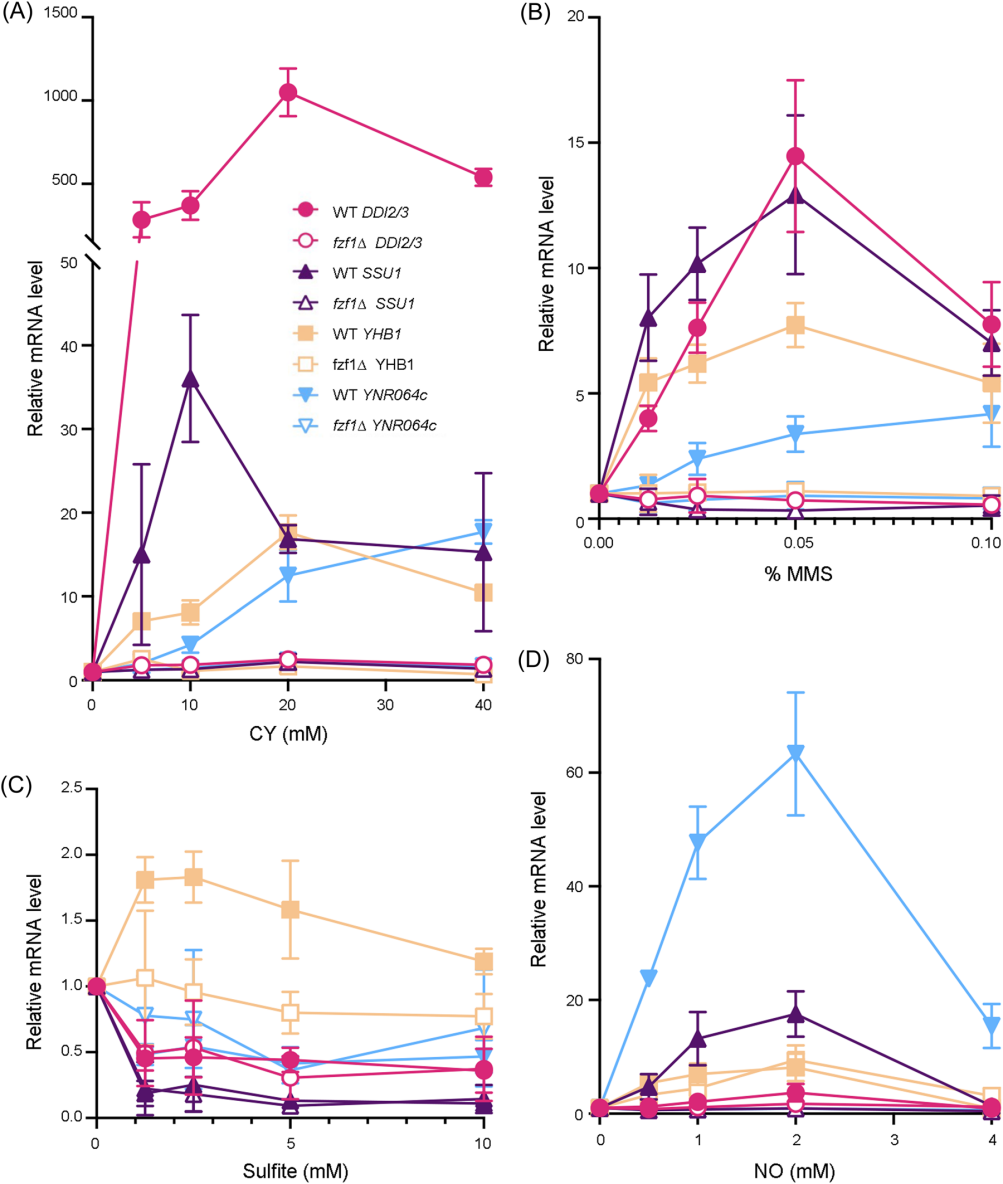
Relative expression of Fzf1‐regulated genes in response to various toxic chemical treatments, as measured by quantitative reverse transcription‐PCR. (A, B) Dose‐response of different strains to cyanamide (CY) (A) and methyl methyl methanesulfonate (MMS) (B) treatments for 2 h. (C) Dose‐response of different strains to sodium sulfite for 2 h in the presence of 75 mM tataric acid (TA). (D) Dose‐response of different strains to nitric oxide (NO) (DPTA NONOate) for 80 min. Information regarding the treatment conditions is summarized in Table S2. The results are the average of at least three independent experiments, with standard deviations shown as error bars. DPTA, dipropylenetriamine.

To achieve optimal induction for each chemical, a combination of numerous experimental conditions was devised in reference to previous reports^3^, ^7^, ^9^, ^16^. Finally, we decided to fix the treatment duration while applying a wide range of chemical treatment concentrations, as described in Table S1. For the CY treatment (Figure 1A), concentrations as low as 5 mM CY could induce *DDI2/3* expression by nearly 500‐fold, and 20 mM CY achieved a more than 1000‐fold induction, which is consistent with our previous report^10^. The next highly induced gene was *SSU1*, with the induction levels as high as 36‐fold by 10 mM CY, while *YHB1* and *YNR064c* were also induced to more than 15‐fold by CY (Figure 1A); 0.05% MMS‐induced *DDI2/3* and *SSU1* to similar levels of 13–15‐fold, albeit much lower than that observed for CY. In comparison, the optimal induction by MMS was sevenfold for *YHB1* and less than fivefold for *YNR064c* (Figure 1B). The *YNR064c* induction curves for both CY and MMS differed from the other genes, in which the highest examined doses resulted in the highest fold induction, indicating that they may have not yet reached maximum induction. As low as 0.5 mM NO could induce *YHB1* by several folds, and the optimal 10‐fold *YHB1* induction by NO is consistent with a previous report^3^. Interestingly, NO treatment induced *YNR064c* by more than 60‐fold and *SSU1* by about 20‐fold. In contrast, *DDI2/3* was barely induced by NO (Figure 1C).

**Figure 2:**
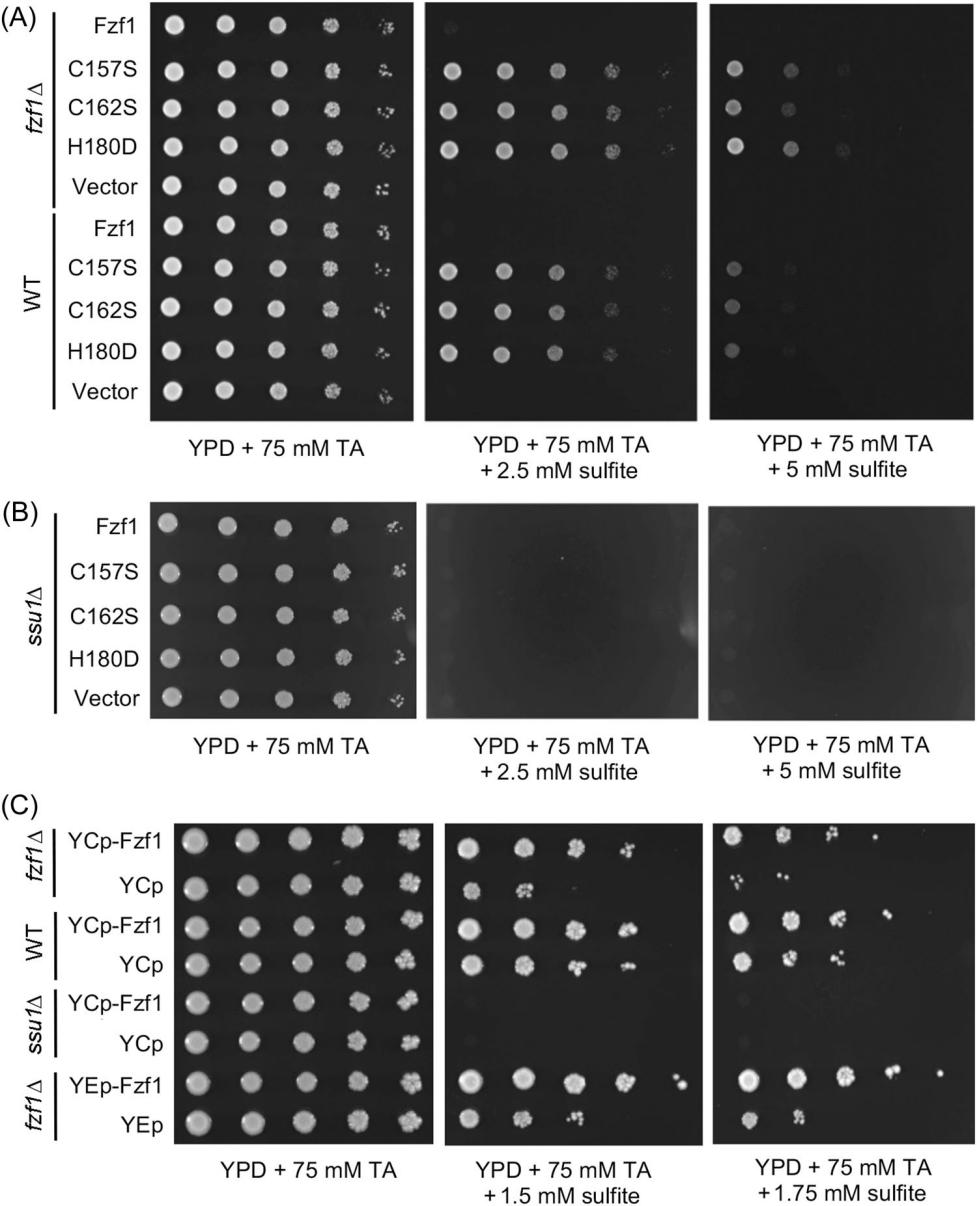
Relative sensitivity of BY4741 and its mutant derivatives to sodium sulfite in the presence of 75 mM TA by serial dilution assays. (A) Plasmids carrying *FZF1* and *fzf1–ZF4* mutations transformed into wild‐type (WT) and *fzf1*∆ cells. (B) Plasmids carrying *FZF1* and *fzf1–ZF4* mutations transformed into the *ssu1*∆ cells. (C) Comparison of relative sulfite sensitivity of cells carrying single‐copy (YCp) and multicopy (YEp) *FZF1* genes. The cells were incubated at 30^°^C for 2 days before photography.

Sodium sulfite treatment alone did not appear to inhibit yeast cell growth (Figure S1A) or affect Fzf1‐regulated gene expression (Figure S1B). It has been reported that most sulfite forms do not exert toxic effects on yeast cells as they cannot penetrate the intracellular environment^20^. In the presence of 75 mM tartaric acid (TA), the culture medium becomes acidic (pH 3.0–4.0)^21^, and sodium sulfite is in sulfurous acid (H_2_SO_3_) form, which can be transported into cells and inhibit yeast cell growth^22^. TA itself did not affect yeast cell growth (Figure 2), but it variably affected Fzf1‐regulated gene expression, particularly for *SSU1* (Figure S1C). Hence, we examined Fzf1‐regulated gene expression in response to sodium sulfite in the presence of 75 mM TA. To our surprise, none of the genes, including *SSU1*, displayed noticeable induction by up to 10 mM sodium sulfite. TA and sulfite treatment reduced *DDI2/3*, *SSU1*, and *YNR064c* expression under our experimental conditions (Figure 1D).

Regardless of the level of induction, deletion of *FZF1* universally abolished the chemically induced expression of all the genes examined (Figure 1). The only exception was *YHB1*, which could still be induced by NO in *fzf1* null mutant cells (Figure 1D), indicating an Fzf1‐independent induction mechanism in this case. Furthermore, deletion of *FZF1* did not relieve sodium sulfite repressed *DDI2/3*, *SSU1*, or *YNR064c* expression (Figure 1D), indicating that repression of these genes by TA and sulfite occurs through an *FZF1*‐independent mechanism.

### *fzf1–ZF4* mutations confer sulfite resistance

Our second hypothesis predicted that yeast cells carrying *fzf1–ZF4* mutations confer sulfite resistance in comparison to wild‐type (WT) cells and that this resistance is due to increased cellular *SSU1* expression. Strains were created by transforming *fzf1*∆ cells with a single‐copy plasmid carrying either *FZF1* or *fzf1*–*ZF4* mutations driven by their native promoters and terminators. All three *fzf1–ZF4* mutants (i.e., *fzf1‐H180D*, *fzf1‐C157S*, and *fzf1‐C162S*) displayed sulfite resistance compared to the vector alone or *FZF1*‐transformed cells in gradient plate (Figure S2) and serial dilution (Figure 2A) assays.

To investigate whether the sulfite resistance conferred by *fzf1–ZF4* mutations is dependent on functional *SSU1*, we created an *SSU1* deletion strain and transformed it with the aforementioned plasmids. As shown in Figure 2B, none of the three *fzf1–ZF4* transformants displayed sulfite resistance under the same experimental conditions. Hence, we conclude that *fzf1* mutations affecting ZF4 function cause sulfite resistance in an Ssu1‐dependent manner.

### *fzf1–ZF4* mutations are codominant with *FZF1*

To further investigate the mechanism of sulfite resistance conferred by *fzf1–ZF4* mutations, we examined whether these *fzf1–ZF4* mutations are dominant or recessive over WT *FZF1*. We transformed WT yeast cells with empty vector, *FZF1* or *fzf1–ZF4* plasmids. As shown in Figures S2 and 2A, *fzf1–ZF4* mutations transformed into WT cells still displayed apparent resistance to sulfite, indicating that these *fzf1–ZF4* mutations are dominant. However, careful examination at higher sulfite concentrations using the gradient plate assay (e.g., 7.5 mM) revealed that *fzf1–ZF4* transformed *fzf1∆* cells are more resistant to sulfite than *fzf1–ZF4* transformed WT cells (Figure S2C). This effect is more obvious in a semi‐quantitative serial dilution assay (Figure 2A, 5 mM sulfite), in which cells containing only an *fzf1*–*ZF4* mutation are approximately 10‐fold more resistant to sulfite than cells carrying both *FZF1* and the *fzf1–ZF4* mutations. Hence, *fzf1–ZF4* mutations are best described as codominant over the WT allele with respect to sulfite resistance.

If the sole effect of *fzf1–ZF4* mutations is to activate Fzf1 in cis, we anticipated that an increased *FZF1* copy number could also confer cellular resistance to sulfite. To test this hypothesis, we examined the cell growth of both YCp single‐copy vector and *FZF1* transformed WT and *fzf1∆* cells in the presence of low doses of sodium sulfite. As shown in Figure 2C, vector‐transformed *fzf1*∆ cells, which lacked *FZF1*, barely grew in the presence of 1.5 or 1.75 mM sulfite. Cells carrying one copy (e.g., WT/vector and *fzf1∆*/*FZF1*) or two copies (e.g., WT/*FZF1*) of *FZF1* displayed increased resistance compared to the *fzf1*∆/vector cells. It appears that cells carrying two copies of *FZF1* are more resistant to sulfite than those carrying one copy of *FZF1* either in a plasmid or on the chromosome and that the resistance is dependent on functional *SSU1*. To further examine the dosage effect, we transformed *fzf*1∆ cells with a YEp multicopy plasmid carrying the *FZF1* gene and found that the transformant was more resistant to sulfite than the cells carrying a single copy or two copies of *FZF1* (Figure 2C).

### *fzf1–ZF4* mutations increase *SSU1* expression

**Figure 3:**
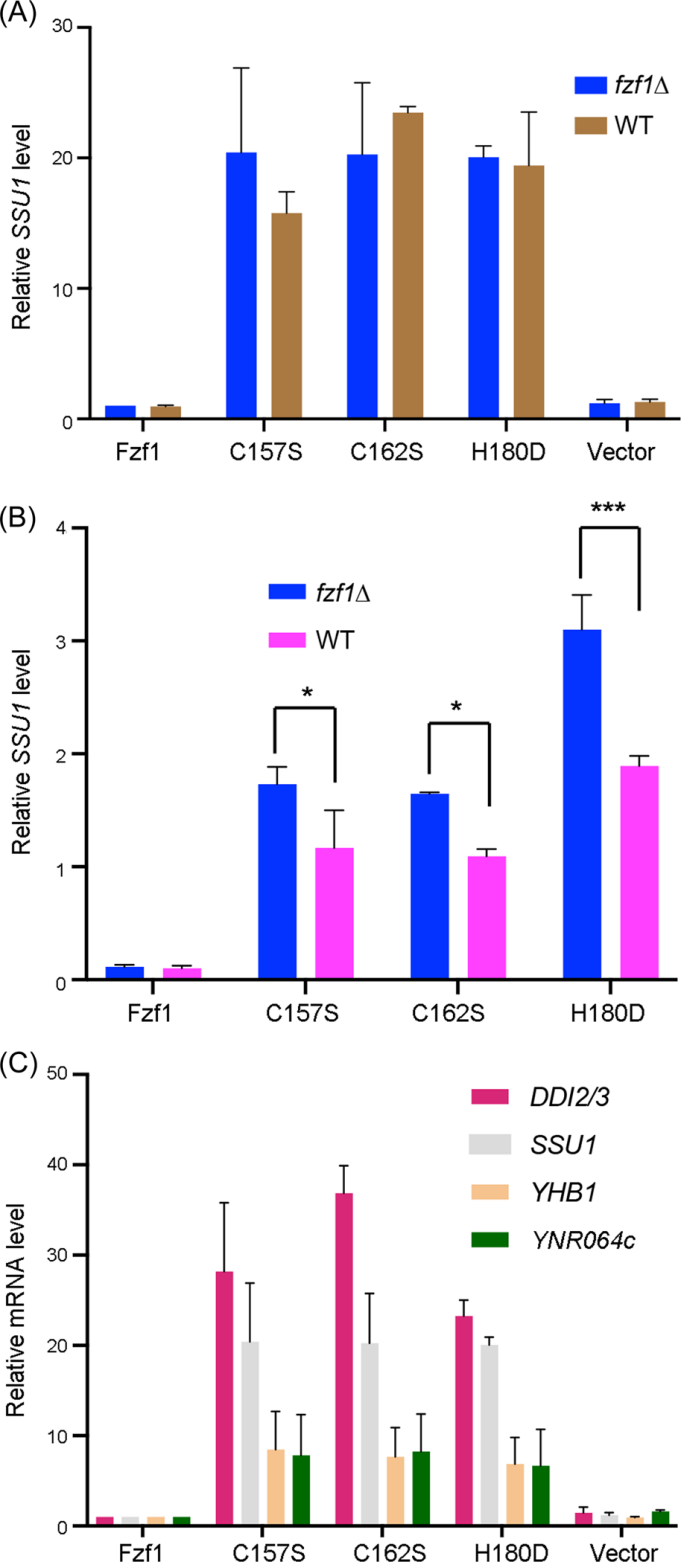
Relative transcript levels of Fzf1‐regulated genes in wild‐type (WT) (BY4741) and isogenic *fzf1∆* cells transformed with plasmids carrying *fzf1–ZF4* mutations. WT or *fzf1∆* cells were transformed with YCp single‐copy plasmids carrying *FZF1* or *fzf1–ZF4* mutations and subjected to a quantitative reverse transcription‐PCR assay. (A) Basal‐level *SSU1* expression in WT and its *fzf1∆* cells transformed with *FZF1* or *fzf1–ZF4* mutations (C157S, C162S, and H180D). (B) Relative transcript levels of *SSU1* in WT and its *fzf1∆* cells transformed with *FZF1* or *fzf1–ZF4* mutations in response to 75 mM TA plus 5 mM sodium sulfite treatment for 2 h. (C) Basal‐level expression of Fzf1‐regulated genes in *fzf1∆* cells transformed with *FZF1* or *fzf1–ZF4* mutations. All values are relative to the corresponding transcript levels in the *fzf1*∆ cells transformed with YCp‐FZF1 without chemical treatment. The data are the average of at least three independent experiments, with standard deviations shown as error bars.

Since the sulfite resistance caused by both *fzf1–ZF4* mutations and the increased *FZF1* gene dosage is dependent on *SSU1*, an obvious prediction is that *fzf1–ZF4* mutations increase *SSU1* expression. To further investigate the relationship between *SSU1* expression and sulfite resistance, transcript levels of *SSU1* in *fzf1–ZF4* mutants, which were assessed by quantitative reverse transcription‐PCR (qRT‐PCR), increased by approximately 20‐fold over WT cells in the absence of chemical treatment (Figure 3A). This is consistent with the notion that ZF4 in Fzf1 functions to negatively regulate transcriptional activation in Fzf1 by default and that this negative regulation is somehow overcome by the chemical modification process. Surprisingly, *FZF1–ZF4* transformed WT cells also exhibited comparable levels of *SSU1* transcript (Figure 3A), which does not explain why *fzf1–ZF4*/*fzf1*∆ cells are more resistant to sulfite than *fzf1–ZF4*/WT cells (Figure 2A).

Cells treated with TA and sodium sulfite exhibited decreased *SSU1* expression under our experimental conditions (Figure 1D). Since the sulfite sensitivity test was conducted in the presence of TA and sodium sulfite, we measured *SSU1* transcript levels using qRT‐PCR under the aforementioned conditions. The *fzf1–ZF4*/*fzf1*∆ cells exhibited higher *SSU1* transcript levels than the *fzf1–ZF4*/WT cells after treatment with TA plus 5 mM sodium sulfite (Figure 3B), consistent with their observed sulfite‐resistant phenotypes.

To investigate whether *fzf1–ZF4* mutations also affect other genes regulated by Fzf1, we measured basal‐level transcripts of *DDI2/3*, *YHB1*, and *YNR064c* in transformed *fzf1*∆ cells. As shown in Figure 3C, in comparison to *FZF1* transformed cells, *fzf1–ZF4* transformed cells displayed an average 30‐fold increase in *DDI2/3* expression, approximately 20‐fold in *SSU1* expression, and around a 10‐fold increase in *YHB1* and *YNR064c* expression, regardless of which ZF4 point mutations were present. In contrast, transcript levels of these genes in the vector‐transformed cells were comparable to *FZF1* transformed cells. These observations collectively indicate that ZF4 serves as an inhibitory component of Fzf1 for all Fzf1‐regulated genes.

### ZF4‐independent induction of Fzf1‐regulated genes

**Figure 4:**
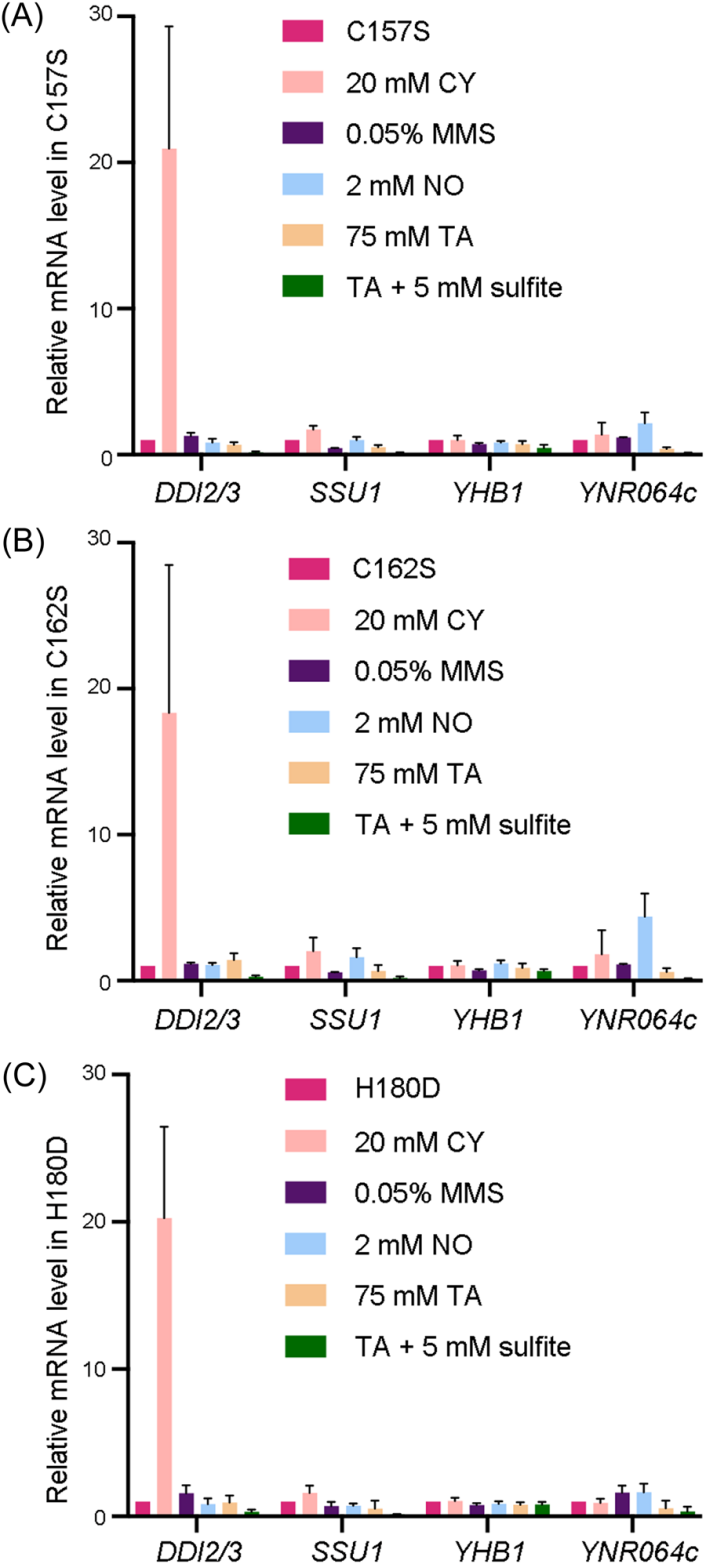
Relative transcript levels of Fzf1‐regulated genes under different chemical treatment conditions. BY4741 *fzf1*∆ cells were transformed with plasmid YCp plasmids carrying *FZF1* or *fzf1–ZF4* mutations and then subjected to a quantitative reverse transcription‐PCR assay. YCplac111 empty vector was also transformed as a negative control. (A) The *fzf1*–C157S mutant. (B) The *fzf1*‐C162S mutant. (C) The *fzf1*‐H180D mutant. The data are the average of at least three independent experiments, with standard deviations shown as error bars.

If chemically induced target gene expression was solely due to ZF4 inhibition of Fzf1 transcriptional activation, we predicted that in the *fzf1–ZF4* mutant cells, these genes are no longer induced by chemical treatment. Indeed, the chemical treatments were no longer able to further induce most Fzf1‐regulated genes in all three *fzf1–ZF4* mutants except *DDI2/3*, which was still induced by CY by approximately 20‐fold (Figure 4). Since deletion of *FZF1* completely abolishes CY‐induced *DDI2/3* expression (Figure 1A), this finding suggests that Fzf1 regulates the *DDI2/3* response to CY by another mechanism independently of ZF4 de‐repression. In addition to the above mentioned CY‐*DDI2/3* combinatorial effect, it appears that NO could also moderately but consistently induce *YNR064c* expression in all three *fzf1–ZF4* mutants (Figure 4). Since *YNR064c* is the most highly induced gene by NO among all Fzf1‐regulated genes, and this induction is completely abolished in *fzf1*∆ cells (Figure 1C), we cautiously conclude that Fzf1 responds to NO‐induced *YNR064c* expression through two separate mechanisms: one is to de‐repress Fzf1 via ZF4, while the other is independent of ZF4.

### ZF5 is required to maintain the Fzf1 function

**Figure 5:**
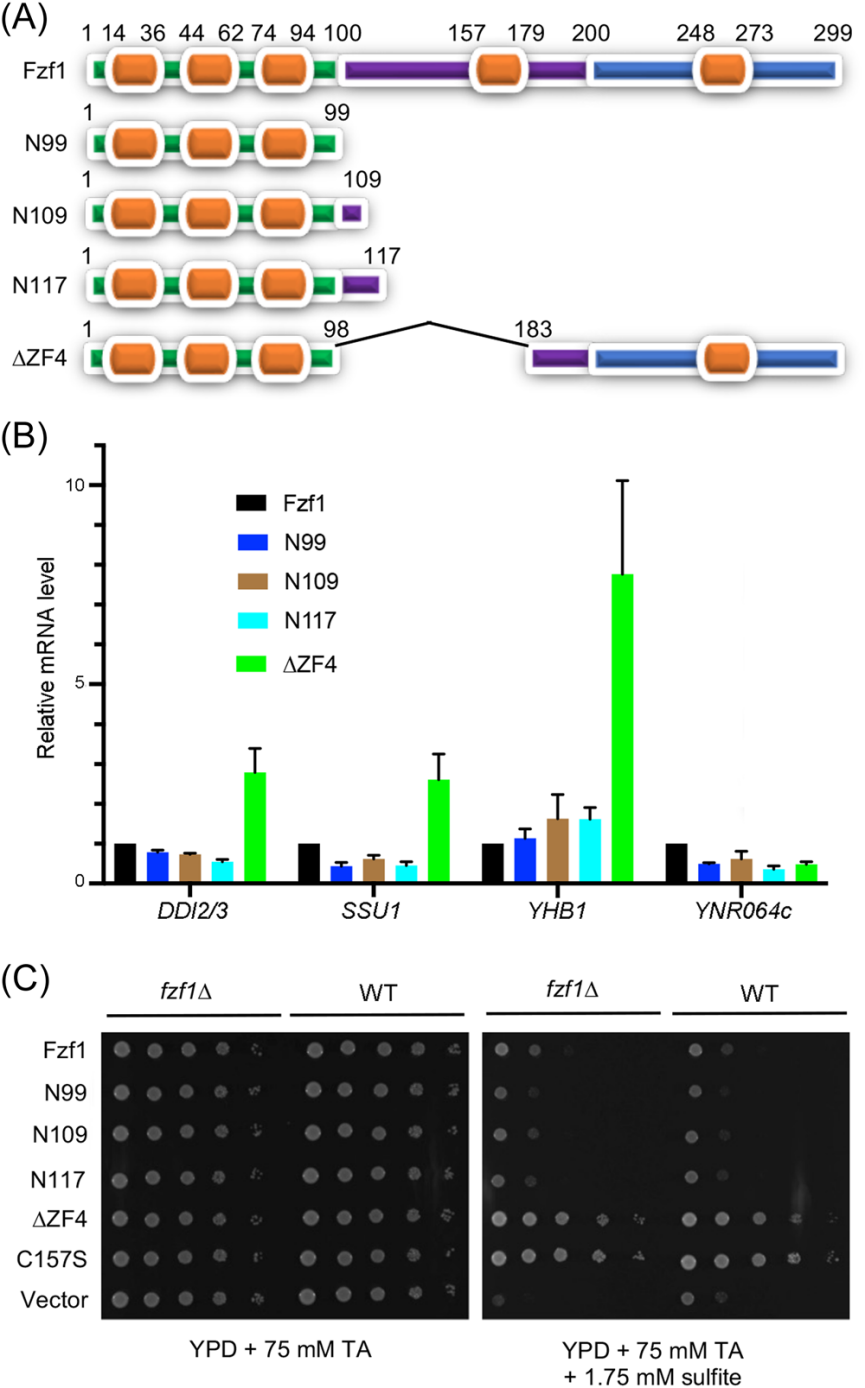
Phenotypes of Fzf1 functional domain truncations. (A) Schematic illustration of different Fzf1 truncations created in this study. The amino acid locations of the ZF domains and truncation sites are indicated. (B) Basal‐level expression of Fzf1‐regulated genes in Fzf1 truncations. WT *fzf1∆* cells were transformed with YCp plasmids carrying various Fzf1 truncations, as indicated, followed by a quantitative reverse transcription‐PCR assay. Values are relative to the *FZF1* gene transformant. The data are the average of at least three independent experiments with standard deviations shown as error bars. (C) Sensitivity of WT and its *fzf1∆* cells transformed with different Fzf1 truncations growing on YPD plates in the presence of 75 mM tataric acid plus 1.75 mM sodium sulfite. The cells were incubated at 30°C for 2 days before photography. Plates containing various sulfite concentrations were tested, and only a representative image is shown.

Since Fzf1 ZF1–3 are sufficient to bind the *SSU1* promoter with nanomolar affinity in vitro^16^, we investigated whether Fzf1 ZF1–3 alone could induce downstream gene expression by making various C‐terminal truncations, as illustrated in Figure 5A, to remove ZF4 and ZF5, and then tested their ability to support target gene expression. None of the three C‐terminal truncations increased basal‐level target gene expression (Figure 5B), nor did they confer sulfite resistance (Figure 5C). In addition, they failed to respond to the chemical induction of Fzf1‐regulated genes, as illustrated by CY‐induced *DDI2/3* expression (Figure S3), probably due to their lack of an activation domain or inability to recruit such a partner activator. However, we cannot rule out another possibility, namely, that these truncated proteins were unstable in yeast cells and hence behaved like *fzf1* null mutants.

We also made an internal truncation construct that produces Fzf1 missing the entire ZF4, designated as Fzf1‐∆ZF4 (Figure 5A). As expected, *fzf1‐∆ZF4* transformed cells increased basal‐level target gene expression except *YNR064c* (Figure 5B) and conferred sulfite resistance (Figure 5C), reminiscent of *fzf1–ZF4* point mutations. Furthermore, *DDI2/3* could still be fully induced by CY in *fzf1‐∆ZF4* cells (Figure S3). Collectively, we conclude that ZF4 is dispensable, while ZF5 is required for Fzf1 function.

### Fzf1 and Fzf1‐∆ZF4 bind target DNA with comparable affinity in vitro

**Figure 6:**
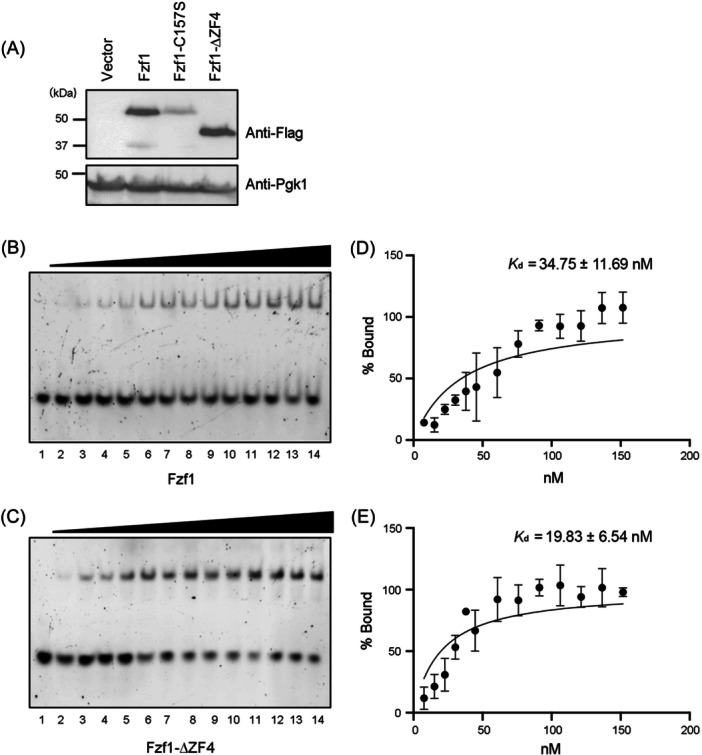
Assessment of Fzf1 mutant derivatives. (A) Western blot analysis of *FZF1* transformants. Yeast whole‐cell extracts carrying plasmids, as indicated in the top panel, were subjected to western blot analysis against anti‐Flag (upper image) or anti‐Pgk1 (lower image) antibodies. (B–E) Sequence‐specific interaction of Fzf1 (B, D) and Fzf1‐∆ZF4 (C, E) with *SSU1*‐CS2 by EMSA. Representative EMSA images show Fzf1 (B) or Fzf1‐∆ZF4 (C) interaction with FITC‐labeled *SSU1*‐CS2. The triangles indicate increasing protein concentrations in lanes 1–14: 0, 7.5, 15, 22.5, 30, 37.5, 45, 60, 75, 90, 105, 120, 135, and 150 nM, as given in the corresponding graphs. (D, E) Quantitative analysis of the Fzf1 (D) or Fzf1‐∆ZF4 (E) binding affinity for the *SSU1*‐CS2 probe. *K*_d_ values were calculated as described in the Materials and Methods section. EMSA, electrophoresis mobility shift assay.

Since cellular Fzf1 levels are positively correlated with its activity and target gene expression, it is possible that ZF4 serves as a protein stability factor and that its deletion or inactivation increases the cellular Fzf1 level to drive target gene expression. To address this possibility, we tagged *FZF1* with its own promoter and terminator sequences in a YCp plasmid and performed a western blot analysis. Figure 6A shows that compared to WT cells, *fzf1*‐C157S transformed cells reduced cellular Fzf1 protein levels, which is consistent with a previous report^19^. In contrast, the *fzf1‐∆ZF4* mutation did not affect the cellular Fzf1 level. These observations effectively rule out the fluctuation in the Fzf1 protein level as an underlying mechanism of Fzf1 inhibition by ZF4.

To further address how ZF4 affects Fzf1 activity, we hypothesized that ZF4 prevents Fzf1 from binding to its cis‐acting target DNA sequence. To test this hypothesis, we performed an electrophoresis mobility shift assay (EMSA) to assess the in vitro DNA binding affinity of Fzf1 for one of its target sequences, *SSU1*‐CS2. Both GST‐Fzf1 and GST–Fzf1–∆ZF4 fusion proteins were overexpressed and purified from bacterial cells. After GST removal and further purification, the recombinant proteins were used in an EMSA. As shown in Figure 6B,C and calculated in Figure 6D,E, both Fzf1 and Fzf1–∆ZF4 effectively bound an FITC‐labeled *SSU1*‐CS2 probe with comparable *K*_d_ values, although deletion of ZF4 appeared to increase Fzf1 affinity for the *SSU1*‐CS2 by nearly twofold. Hence, the deletion of ZF4 does not significantly affect Fzf1 interaction with its DNA recognition sequence.

## DISCUSSION

This study systematically assessed yeast cellular responses to chemically induced Fzf1‐regulated gene expression. Fzf1 coordinately regulates a group of seemingly unrelated genes whose products function in different toxic stress response pathways. Previous studies have focused on specific gene–chemical pairs, such as *SSU1*‐sulfite^4^, *YHB1*‐NO^3^, and *DDI2/3*‐CY/MMS^12^. This study found that these genes can be cross‐induced by different chemicals in an Fzf1‐dependent manner, although the gene products may not contribute to the chemical detoxification. This phenomenon is not unprecedented; for example, DNA damage induces a group of genes whose products may or may not be involved in the repair of DNA adducts induced by this type of damage^23^. Similarly, a methylating agent, MMS, can induce a large number of genes in budding yeast^24^, ^25^, and most of them do not appear to be involved in cellular responses to MMS toxicity. It is speculated that the coordinated transcriptional regulation of genes may confer an evolutionary advantage. In the case of DNA damage response, a few well‐studied target genes are coordinately regulated by a common checkpoint pathway as sensor and signal transducers^26^, ^27^, ^28^, ^29^, and their effectors or transcriptional regulators are different^26^, ^30^, ^31^. Here, a few diversely functional genes share an effector, Fzf1, in response to chemical toxicity. Whether and how Fzf1 senses different chemical stresses and selects target gene expression is unclear. Nevertheless, a few paired chemical–gene‐specific inductions were observed in this study. For example, CY specifically induced *DDI2/3* by up to 1000‐fold but induced other genes by 20–30‐fold, while NO‐induced *YNR064c* by more than 60‐fold but barely induced *DDI2/3*.

**Figure 7:**
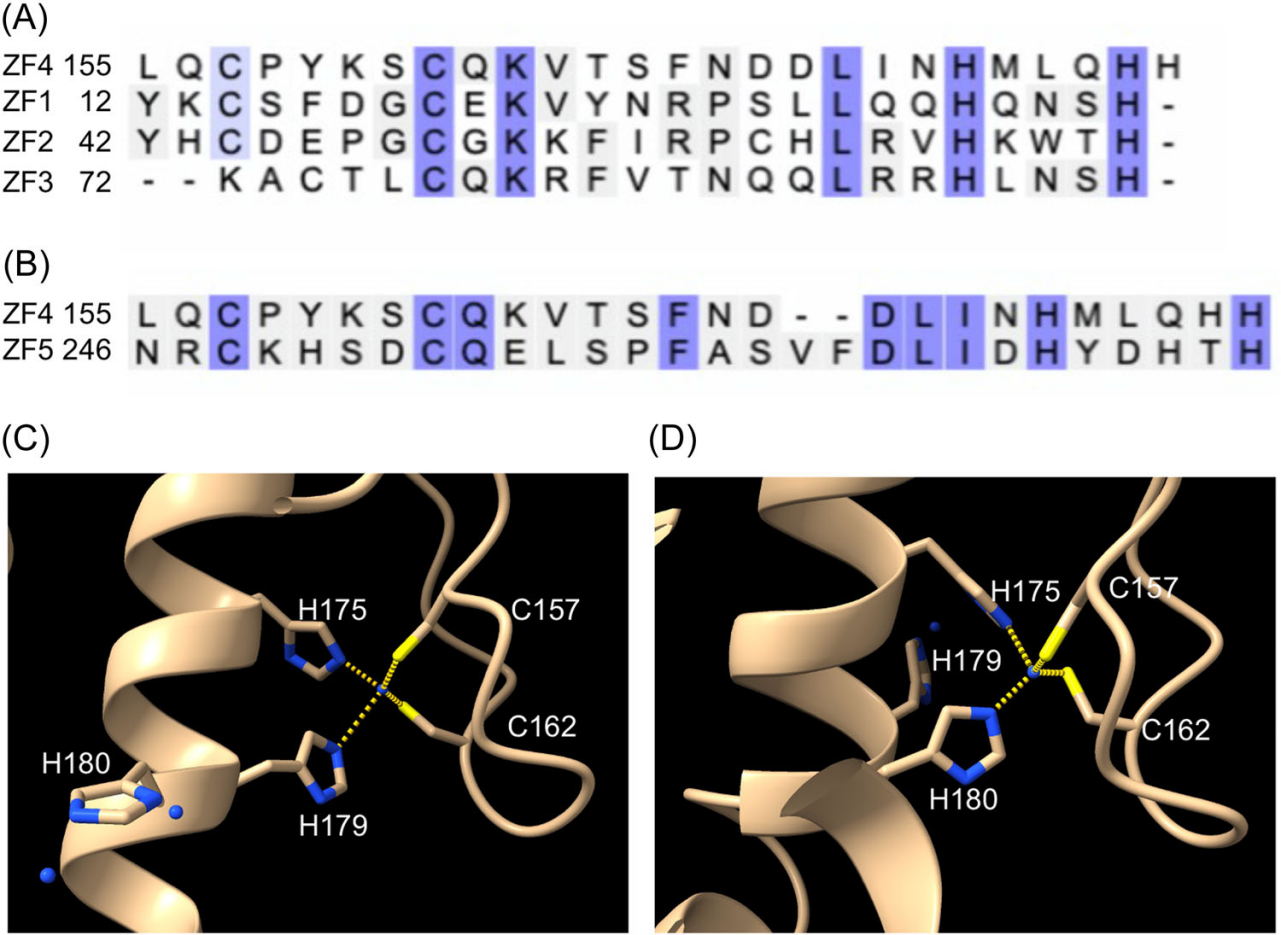
Amino acid sequence alignments and three‐dimensional (3D) structure predictions of Fzf1–ZF4. (A) Amino acid sequence alignment of ZF1–4 in Fzf1. (B) Amino acid sequence alignment of ZFs 4–5 in Fzf1. (C) The 3D structure prediction of Fzf1–ZF4 consisting of C157, C162, H175, and H179. (D) The 3D structure prediction of Fzf1–ZF4 consisting of C157, C162, H175, and H180. The 3D structures (C, D) were generated using the AlphaFold Protein Structure Database.

Several observations from this study support the notion that the ZF4 domain of Fzf1 is a component that inhibits Fzf1 activity. First, C‐to‐S amino acid substitutions on the two ZF4 consensus residues, known to disrupt zinc coordination and hence its secondary structure, resulted in increased expression of all Fzf1‐regulated genes. Second, the Fzf1‐H180D substitution also behaved like the C‐to‐S mutations. However, it is unclear whether H180 is a zinc coordination residue since in a survey of recently reported C_2_H_2_ ZFs, the vast majority (38/41) have three nonconserved residues between the two His residues^32^, while the remaining four variable residues are primarily atypical ZFs^33^, ^34^, ^35^. Interestingly, Fzf1 ZF4 can align with ZF1–3 (Figure 7A) and ZF5 (Figure 7B) in Fzf1 via H179 and H180, respectively, and the AlphaFold Protein Structure Database predicts alternative structures with either H179 or H180 as ZF consensus residues (Figure 7C,D). Although our experimental data support H180 as a zinc coordination residue, we cannot rule out a possibility that H180 plays a different but also critical role in ZF4. Third, a corresponding Fzf1‐∆ZF4, in which the entire ZF4 domain was deleted, resulted in phenotypes reminiscent of *fzf1–ZF4* point mutations. Therefore, the entire ZF4 is not only dispensable for Fzf1 activity, but its removal also makes Fzf1 hyperactive in the absence of chemical inducers. We also ruled out the possibility that ZF4 inactivation serves to stabilize Fzf1, leading to an increased cellular Fzf1 level. Together with a previous report that chemical stresses do not increase the cellular Fzf1 level^12^, we can safely conclude that the chemical activation of Fzf1 is achieved by derepression of its intrinsic ZF4 inhibition.

This study utilized sulfite resistance as an independent assay to examine whether ZF4 is an inhibitor. It has been reported that sodium sulfite‐induced yeast *SSU1* expression^4^, and an *SUL1* mutation *fzf1*‐H180D have been thought to constitutively enhance *SSU1* expression, resulting in sulfite resistance^18^. However, careful examination revealed that *SSU1* induction by sodium sulfite varied among *Saccharomyce* species, and the optimal induction in *S. cerevisiae* was threefold. Furthermore, the study in question did not add TA to the culture medium^4^. Because under our experimental conditions, TA and sulfite treatment inhibited *SSU1* expression by up to 10‐fold, while *fzf1–ZF4* mutations increased *SSU1* transcript levels by 20‐fold over the *FZF1* cells, we infer that the sulfite resistance conferred by *SUL1/fzf1–ZF4* mutations was not due to constitutive activation of *SSU1*, but rather the compensation of reduced *SSU1* levels in the WT cells. This study also carefully assessed the dosage effects of *FZF1* on *SSU1* expression. We conclude that an increased cellular *FZF1* gene copy corresponds to the enhanced *SSU1* expression and levels of sulfite resistance, consistent with previous observations that overexpression of *FZF1* alone is sufficient to confer sulfite resistance^22^ and activate its target gene expression^3^.

The two major activities of a typical transcriptional activator are sequence‐specific DNA binding and transcriptional activation^36^, ^37^. To address how ZF4 inhibits Fzf1 activity, we investigated whether the deletion of ZF4 significantly enhanced its interaction with the target promoter sequence. It has been previously reported that the three N‐terminal ZFs are required and sufficient to bind a DNA fragment containing *SSU1* promoter sequence −506 to −315, and a DNase I protection assay revealed a footprint between nucleotides −442 and −420^16^. However, subsequent studies indicated that Fzf1 probably binds a distinct consensus sequence CS2 (5′‐NGNNNNCTATCANTTNN‐3′) found in all known Fzf1‐regulated gene promoters^3^, ^12^. This study used a 27‐bp CS2 probe from the*SSU1* promoter corresponding to nucleotides −392 to −366 and found that both Fzf1 and Fzf1–∆ZF4 bind the *SSU1*‐CS2 probe with comparable affinity in vitro, supporting the notion that although both Fzf1 and its Fzf1–ZF4 mutant derivatives can bind the *SSU1* promoter, only ZF4 mutated Fzf1 can effectively activate the target gene expression in the absence of a chemical inducer. Since Fzf1 can compete with Fzf1–ZF4 mutants for the promoter binding in *FZF1/fzf1–ZF4* cells, it explains our observation that *fzf1–ZF4* mutations and *FZF1* are codominant and rules out the possibility that ZF4 inhibits Fzf1 activity by preventing it from forming an Fzf1–CS2 interaction.

In summary, this study addressed our two initial hypotheses, as discussed above. However, at least two additional questions arose regarding Fzf1 functions. First, although it is clear that the chemical activation of Fzf1 is due to derepression, it remains unclear what activates Fzf1. Fzf1–ZF5 is unnecessary for target promoter binding^16^, and this study revealed that ZF5 is required for Fzf1 activation. However, this region does not contain a known transcriptional activation motif, and whether it functions as an activation domain or by recruiting another activator requires further investigation. Alternatively, residues in Fzf1 may be modified upon chemical stress to overcome ZF4 inhibition through conformational change, turning it into an activator^12^, ^38^. Second, while most Fzf1‐regulated genes are not further induced by chemical treatments in *fzf1–ZF4* mutant cells, CY can still induce *DDI2/3* by another 20‐fold. This Fzf1‐dependent but ZF4‐independent activity requires further investigation. In this regard, it is interesting to note that in a genome‐wide study of the gene expression profile after histone H4 depletion^39^, *DDI2* and *DDI3* were the most highly induced genes. Hence, it seems possible that Fzf1 may interact with nucleosomes to regulate *DDI2/3* expression. A protein BLAST search of the genomic database revealed that Fzf1 is only conserved within fungal species, and its closely related homolog has not been found in plants and animals^32^. Further investigation is needed to address whether negative regulation by a ZF domain is a common phenomenon.

## MATERIALS AND METHODS

### Yeast strains and culture

All haploid yeast strains used in this study were derived from BY4741 (*MAT**a** his3∆1 leu2∆0 met15∆0 ura3∆0*). The isogenic *fzf1∆::KanMX4* and *ssu1∆::KanMX4* mutants were generated by the *Saccharomyces* Genome Deletion Project Consortium and purchased from Research Genetics, in which the entire target gene open reading frames (ORF) were deleted and replaced by a *kanMX* selectable marker by a one‐step gene disruption method^40^. Yeast cells were incubated in a yeast extract‐peptone‐dextrose (YPD) or synthetic dextrose (SD) medium supplemented with appropriate nutrients, as previously described^41^.

### Plasmid construction and site‐specific mutagenesis

A 1.7‐kb yeast genomic DNA fragment containing the entire *FZF1* ORF along with its 0.5‐kb promoter sequence and 0.3‐kb terminator sequence was inserted into single‐copy vector YCplac111 and multi‐copy vector YEplac181^42^ as described^12^ to form YCpL–Fzf1 and YEpL–Fzf1, respectively.

Desired *fzf1*–*ZF4* mutations were created by site‐directed mutagenesis using a modified QuickChange method^43^. To make *fzf1–ZF4* mutations or Fzf1 truncations, plasmid YCpL–Fzf1 was amplified by PCR using mutagenic primers or designed truncation overlap primers (Table S2). After digestion with *Dpn*I to remove the template DNA, the PCR product was transformed into *Escherichia coli* DH10B cells to screen for the targeted mutations and truncations. The entire *FZF1* inserts containing the desired mutations or truncations were confirmed by sequencing.

To assess cloned Fzf1 protein levels in transformed yeast cells, 3xHA, 3xFlag, and His_6_ tag^44^ were inserted into YCpL–Fzf1 at the C‐terminus to form YCpL–Fzf1–HFH. Desired *fzf1* mutant derivatives were then used to replace the wild‐type *FZF1*.

### Yeast cell transformation

Yeast cells were transformed with plasmids using a lithium acetate method^45^ as described^46^.

### Yeast survival assays

Sulfite resistance was assessed using both serial dilution and gradient plate assays as described^47^. To make serial dilution plates, autoclaved YPD agar medium was cooled to approximately 55°C, supplemented with 3 M TA stock solution to a final concentration of 75 mM and 1 M sodium sulfite stock solution to final concentrations as indicated before pouring plates. To make gradient plates, the lower layer of YPD agar contained 75 mM TA and various concentrations of sodium sulfite, while the upper layer contained YPD agar only.

### Yeast RNA extraction and qRT‐PCR

Yeast cells were initially cultured in YPD or SD medium at 30°C overnight. The cultures were then inoculated again and grown until reaching an OD_600 nm_ of approximately 0.2–0.3, which typically took around 2 h. Subsequently, the cells were treated with various chemicals for specific durations: CY, MMS, and sodium sulfite+TA treatments were for 2 h, while the NO treatment was for 1.5 h. Untreated cells were incubated for an additional 2 h.

The cells were harvested by centrifugation and then subjected to lysis using 200 U zymolyase (Amsbio, Cat. 120491‐1) per 5 × 10^7^ yeast cells for 1 h. Total RNA was extracted from the lysed cells using a yeast RNA extraction kit (RBY300; Geneaid). Extracted RNA samples were reverse‐transcribed into cDNA, and the expression levels of the tested genes were assessed using qPCR with iQ^TM^ SYBR Green Super mix (Cat. 170‐8882; Bio‐Rad). Data analysis was performed using the $2^{-∆∆\mathrm{CT}}$ method, as described^48^ to determine relative expression levels of genes of interest relative to a reference gene. The internal control gene *UBC6* was selected for normalization as previously justified^12^.

### Western blot analysis

Yeast whole‐cell extracts (WCEs) were prepared from BY4741 *fzf1∆::KanMX4* cells transformed with plasmid YCpL‐Fzf1‐HFH and its mutant derivatives and subjected to western blot analysis against an M2 anti‐Flag antibody (Cat. F1804, 1:5000 dilution; Sigma) as previously described^12^. An anti‐Pgk1 polyclonal antibody received from Dr. W. Li (Institute of Zoology, Chinese Academy of Sciences) was used as a loading control.

### Recombinant Fzf1 protein production and purification

The *FZF1* ORF was cloned into plasmid pGEX‐6P‐1 to produce an N‐terminal GST fusion protein. The corresponding ∆ZF4 truncation was made by a method described earlier using the resulting pGEX‐Fzf1 as a template. These plasmids were transformed into *E. coli* Rosetta cells to overexpress recombinant proteins. After IPTG induction, the cells were lysed and genomic DNA was treated with benzonase nuclease (Cat. 11442587; Thermo Scientific) to eliminate nonspecific DNA binding by the recombinant GST‐Fzf1, followed by two‐rounds of purification using GST beads (Glutathione Sepharose^TM^ 4B, Cat. 45‐002‐065; Cytiva) and one round of purification using heparin beads (Heparin Sepharose^TM^ 6 Fast Flow, Cat. 17099801; Cytiva). After the PreScission protease cleavage and GST affinity chromatography to remove GST, the resulting Fzf1 protein purity and concentration were determined.

### EMSA

Each 20‐µl EMSA reaction contained 0.1 pmol dsDNA probe, which was made by annealing an unlabeled DNA strand with a fluorescein isothiocyanate (FITC)‐labeled strand (Table S2), 2 ng bovine serum albumin, and 0.5 µg poly dI‐dC (Cat. 11430605; Thermo Scientific) in a reaction buffer. After 20 min incubation on ice, the mixtures were subjected to 6% native polyacrylamide gel electrophoresis and imaged by fluorescence of FITC at 488 nm.

The *K*_d_ value in this experiment shows a half‐binding concentration of protein to a constant amount of DNA probe. To estimate the value, EMSA results were used to measure the intensity of both protein‐DNA binding bands and free probe bands by Adobe Photoshop, and the binding was calculated from the shifted band intensity using an equation: binding intensity/(binding intensity + free intensity) × 100%. In the EMSA assay, the binding would not change when reaching the highest binding ability among increased protein concentrations, which is also considered as a standard saturated titration. The binding percentage in each protein concentration was calculated by the equation: binding rate/saturated titration × 100%. The binding curves were generated with GraphPad Prism using an equation: *“*[Agonist] versus normalized response” [binding percentage = 100 × protein concentration/(*K*_d_ + protein concentration)].

### Protein structure prediction

The Fzf1 full‐length and Fzf1–∆ZF4 protein structures and zinc ion positions were predicted by running Metal 3D CNN^49^ AlphaFold^50^ or online AlphaFold Protein Structure Database^51^, ^52^, and the data were provided by the AFDB Clusters^53^.

## AUTHOR CONTRIBUTIONS

**Ying Du**: Conceptualization (equal); data curation (equal); formal analysis (equal); writing—original draft (equal); writing—review and editing (equal). **Chaoqun Ma**: Formal analysis (equal); methodology (equal). **Stanley A. Moore**: Formal analysis (equal); supervision (equal); writing—review and editing (equal). **Wei Xiao**: Conceptualization (equal); formal analysis (equal); funding acquisition (equal); project administration (equal); supervision (equal); writing—original draft (equal); writing—review and editing (equal).

## ETHICS STATEMENT

The study in this article did not involve any trials on humans or animals.

## CONFLICT OF INTERESTS

The authors declare no conflict of interests.

## Supporting information

Supporting information.

## Data Availability

The data set generated during and/or analyzed during the current study is available from the corresponding author upon reasonable request.
