# Supplementary material for: Zinc finger 4 negatively controls the transcriptional activator Fzf1 in Saccharomyces cerevisiae
Source: mLife. 2024 Sep 23;3(3):391–402. doi: 10.1002/mlf2.12141 (PMC11442136; doi:10.1002/mlf2.12141)
Supplement: Supplementary file 1 — Supporting information. [file MLF2-3-391-s001.docx]

**Table S1.** Chemical treatment conditions in literature and employed in this study

|  | **Cyanamide** | **Sodium sulfite** | **DPTA NONOate** | **MMS** |
| --- | --- | --- | --- | --- |
| **Doses used in literature** | 0 - 40 mM | < 1 mM | 0.1 - 1 mM | 0.01 - 0.05% |
| **Duration in literature** | 2 hours | < 1 hour | Up to 80 min | 2 hours |
| **Doses used in this study** | Up to 40 mM | Up to 10 mM (with 75 mM TA) | Up to 4 mM | Up to 0.1% |
| **Duration used in this study** | 2 hours | 2 hours | 80 min | 2 hours |

Cyanamide data come from (Li et al., 2015); sodium sulfite data come from (Engle and Fay, 2012); DPTA NONOate data come from (Sarver and DeRisi, 2005); MMS data come from (Lin et al., 2023).

**Table S2.** Oligonucleotide sequences used in this study

| **Name** | **Sequence (5’-3’)** |
| --- | --- |
| Fzf1-C157S-F | CATGCAAGTGCTACAAAGCCCATACAAATCATGTC |
| Fzf1-C157S-R | GACATGATTTGTATGGGCTTTGTAGCACTTGCATG |
| Fzf1-C162S-F | GCCCATACAAATCAAGTCAAAAAGTTACCAGC |
| Fzf1-C162S-R | GCTGGTAACTTTTTGACTTGATTTGTATGGGC |
| Fzf1-H180D-F | CATATGTTGCAACATGATATAGCAAGTAAGC |
| Fzf1-H180D-R | GCTTACTTGCTATATCATGTTGCAACATATG |
| Fzf1-N99-F | CATTTAAATAGTCATGAAAGAAAAAGCAAGTGAACGCCATAGAAGAGCAATTTCCGTCCT |
| Fzf1-N99-R | GAGGACGGAAATTGCTCTTCTATGGCGTTCACTTGCTTTTTCTTTCATGACTATTTAAATG |
| Fzf1-N109-F | CTTGCATCAAGAATTGACCGTAAACACGAATGAACGCCATAGAAGAGCAATTTCCGTCCT |
| Fzf1-N109-R | GAGGACGGAAATTGCTCTTCTATGGCGTTCATTCGTGTTTACGGTCAATTCTTGATGCAAG |
| Fzf1-N117-F | CACGAAGGAGTGAATGCGAATGTGAAAGCATGAACGCCATAGAAGAGCAATTTCCGTCCT |
| Fzf1-N117-R | GAGGACGGAAATTGCTCTTCTATGGCGTTCATGCTTTCACATTCGCATTCACTCCTTCGTG |
| Fzf1-∆ZF4-F | GGCATTTAAATAGTCATGAAAGAAAAAGCAGTAAGCTTGTTGTACCATCTGGAGATCCA |
| Fzf1-∆ZF4-R | GGATCTCCAGATGGTACAACAAGCTTACTGCTTTTTCTTTCATGACTATTTAAATGCC |
| SSU1-CS2-F | CTTCCTGCAAACTATCATTTTTTTTTC |
| SSU1-CS2-R | GAAAAAAAAATGATAGTTTGCAGGAAG |

**Figure S1.** Cellular responses to tartaric acid and sodium sulfite treatments. (A) A serial dilution assay to assess cell growth in response to sulfite. BY471 *fzf1∆* cells were transformed with either YCplac111 vector or YCpL-FZF1 and incubated in YPD plates with or without 5 mM sodium sulfite for 2 days before photography. (B) Relative transcript levels of Fzf1-regulated genes in wild-type BY4741 and its *fzf1∆* cells treated with 5 mM sodium sulfite alone for 2 hours by a qRT-PCR assay. (C) Relative transcript levels of Fzf1-regulated genes in wild-type BY4741 and its *fzf1∆* cells treated with 75 mM tartaric acid (TA) alone for 2 hours by a qRT-PCR assay. All values in (B) and (C) are relative to wild-type BY4741 cells without treatment, and data are average of at least three independent experiments with standard deviations shown as error bars.

**Figure S2.** Relative sensitivity of wild-type BY471 and its *fzf1∆* mutant cells transformed with plasmids containing *FZF1* or *fzf1-ZF4* mutants by a gradient plate assay. (A) No sodium sulfite. (B) 5 mM sodium sulfite gradient. (C) 7.5 mM sodium sulfite gradient. Cells were incubated at 30^o^C for 2 days before photography. Arrows point to increasing sodium sulfite concentrations. Only representative plates containing informative concentrations of sodium sulfite are shown.

**Figure S3.** Relative *DDI2/3* transcript levels in BY4741 *fzf1∆* cells transformed with FZF1 or its truncation derivatives in response to 20 mM CY treatment for 2 hours by a qRT-PCR assay. All values are relative to *fzf1*∆ cells transformed with YCpL-FZF1 without CY treatment. Data are average of at least three independent experiments with standard deviations shown as error bars.


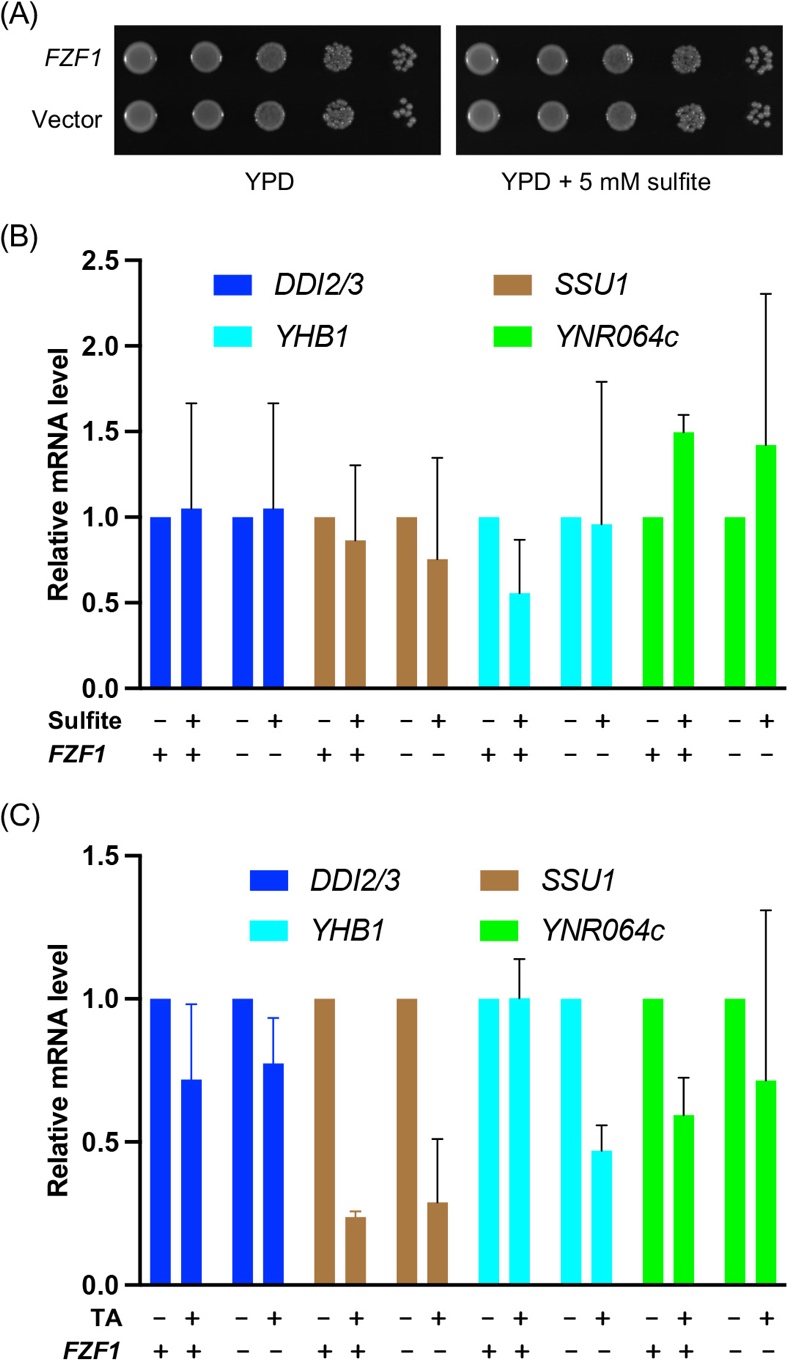


Figure S1


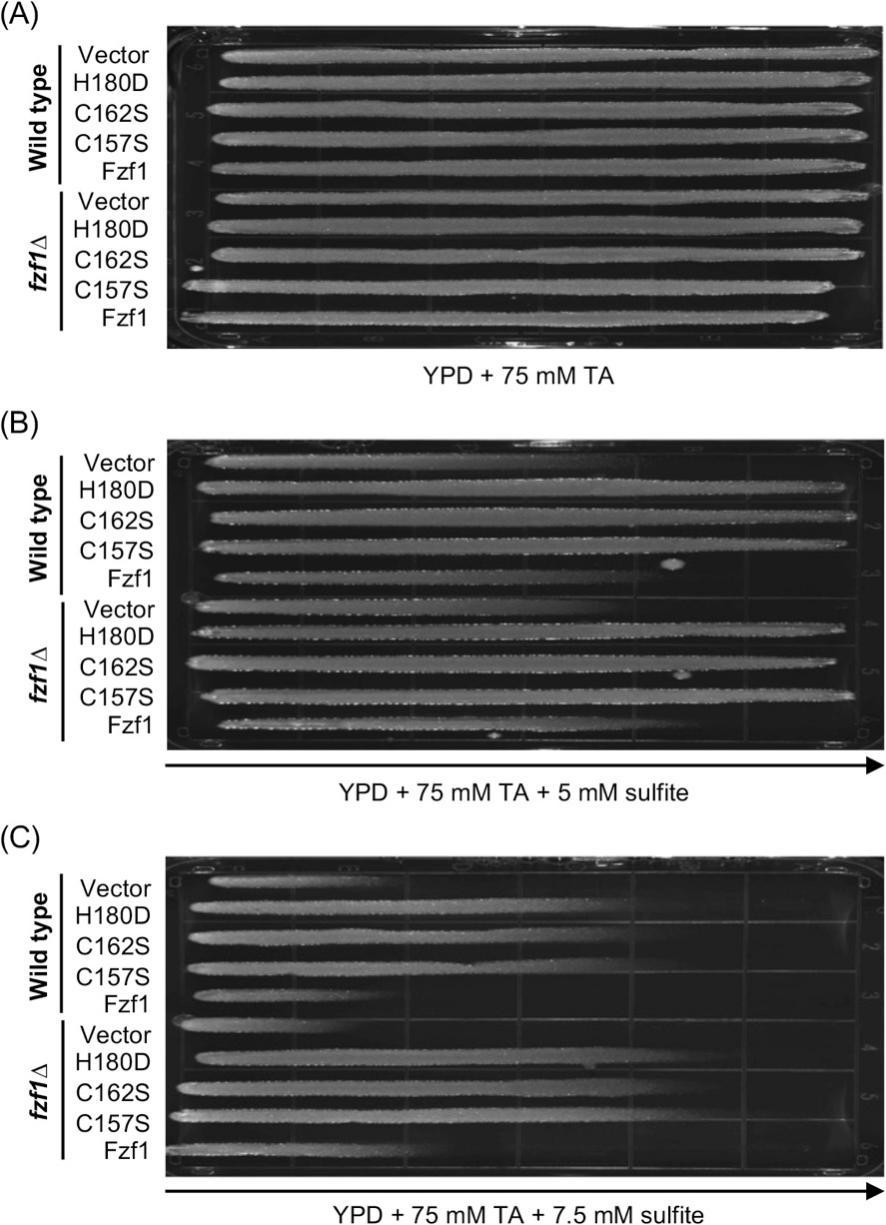


Figure S2


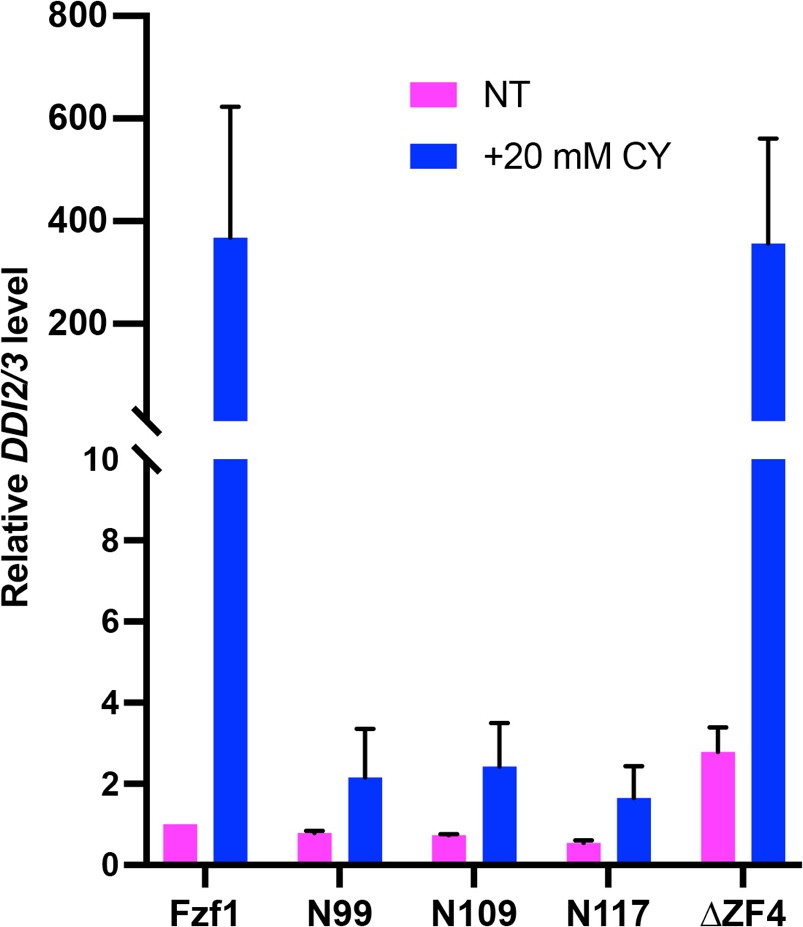


Figure S3
